# A talented giant: a tribute to the memory of John M. Opitz

**DOI:** 10.1186/s13052-024-01711-z

**Published:** 2024-08-07

**Authors:** Lorenzo Pavone, Giovanni Corsello, Martino Ruggieri

**Affiliations:** 1 Pediatric Clinics, Catania University, Catania, Italy; 2 Division of Pediatrics, Di Cristina Benfratelli, “A.R.N.A.S,” Civic Hospital, Palermo University, Palermo, Italy; 3 Section of Pediatrics and Child Neuropsychiatry, Department of Child and Experimental Medicine, Catania University, Catania, Italy

**Keywords:** John Opitz, Genetics, Pediatrics, Sicily, Smith-Lemli-Opitz, SLO syndrome

## Abstract

**Background:**

John M. Opitz, a towering figure in both stature and scientific achievement, left an indelible mark on the fields of genetics, pediatrics, and embryology. Born in 1935 in Hamburg to a Jewish family, Opitz’s early life was marked by adversities. Despite these challenges, he pursued a remarkable career, immigrating to the United States at 15 years and becoming a renowned scientist in institutions like Iowa State University and the University of Wisconsin, where he made groundbreaking contributions to clinical genetics.

**Main body:**

A testament to his compassionate nature, Opitz dedicated himself to understanding and treating rare genetic disorders, earning him eponymous recognition in several medical conditions. His impact extended beyond academia, as evidenced by his collaborative efforts with Sicilian universities to advance clinical genetics in Italy. Opitz’s teaching style emphasized simplicity, empathy, and meticulous clinical examination, leaving an indelible mark on students and colleagues.

**Conclusion:**

John M. Opitz’s towering intellect, compassionate demeanor, and profound impact on medicine and genetics made him a figure of enduring significance. His legacy lives on through the countless lives he touched, the knowledge he transmitted, and the enduring friendships he forged. In remembering John Opitz, we honor not only a man, but also a myth—a symbol of resilience, humanity, and scientific excellence.

## Background

Anyone who had the opportunity to meet John Opitz could not forget his distinctive physical appearance and his humanistic personality. His considerable height and his severe yet reassuring demeanor concealed a troubled youth and the sorrow of a life marked by unfortunate and tragic events. John M. Opitz, an extraordinary figure in science and medicine, was a formidable presence in the fields of genetics, pediatrics, and embryology. His life, characterized by personal challenges and tragic family events, shaped an exceptional individual whose scientific and humanistic contributions have left an indelible mark on the academic and medical community. In this article, we will explore Opitz’s rich and significant journey, from his turbulent past in Hamburg to his remarkable career in the United States and beyond.

## Main text

### The extraordinary scientific attitude

John M. Opitz was born in Hamburg in 1935 into a Jewish family. His father passed away when he was just 5 years old. Due to his poor health and pulmonary infection, he spent considerable time in hospitals and in a sanatorium in Bavaria. During his youth, he lived with his grandfather, and his schooling was particularly challenging due to his fragile health. At the age of 15, he immigrated to the United States, settling initially in Iowa City, Iowa, where he attended high school with a special interest in zoology. At the age of 23, he obtained a bachelor’s degree in zoology from this university. Continuing his studies at Iowa State University, he supported himself by working as a supervisor in the animal room for biomedical experimentation. During his time in Iowa City, he collaborated with Jacqueline Noonan, a pediatric cardiologist known for the syndrome named after her, and later, at the University of Wisconsin, with Professor Klaus Patau, a renowned American cytogeneticist of German descent. During his tenure at this institution, he established the Wisconsin Clinical Genetics Center, as well as the American College and American Board of Medical Genetics. In 1976, he founded and became the Editor-in-Chief of the prestigious American Journal of Medical Genetics. Invited by Professor Philip D. Pallister, an expert in human genetics, he relocated to Helena, Montana, as the Director of the Shodair-Montana Regional Genetic Service Program. John Opitz continued his active career, working at the Hanseatic University Foundation in Lubeck, Germany, as a professor of Pediatrics in Utah, and even spending a sabbatical year in Italy [1]. In 1999, upon the invitation to the National Congress of the Italian Society of Pediatrics, and at the proposal of Professor Gianpaolo Salvioli, already President of the Italian Society of Pediatrics (SIP), he was awarded an honorary Doctorate in Medicine by the Alma Mater Studiorum, University of Bologna.

He dedicated his time to working with children affected by rare clinical disorders, and he disseminated the results of his insightful clinical observations through numerous papers, textbook chapters, editorials, and books. Some of his original clinical reports have been honored with his name, such as Opitz syndrome, or in association with other scientists, as seen in the Opitz-Kaveggia syndrome, Smith-Lemli-Opitz syndrome, Herrman-Opitz syndrome, and Bohring-Opitz syndrome. He is widely acknowledged as one of the foremost scientists in the fields of embryology, pediatrics, genetics, congenital malformations, and rare syndromes.

### The visits to sicilian universities

**Fig. 1:**
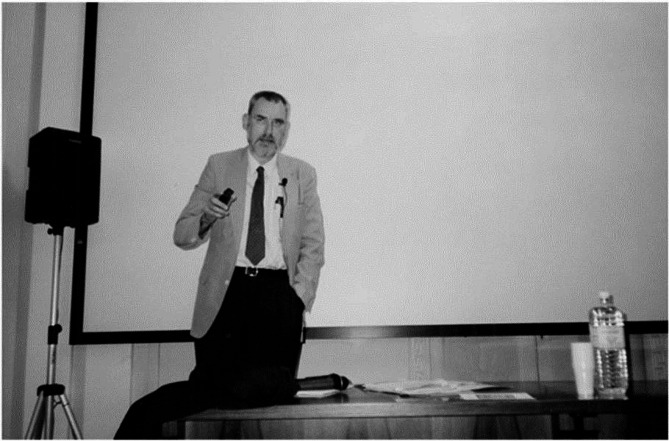
John Opitz in Catania during a lesson to the medical students at the local University

With the friendly collaboration of Prof. Giovanni Neri, a prestigious geneticist at the Catholic University of Rome, the three state universities of Palermo, Catania, and Messina, along with the participation of the IRCCS Association OASI Maria SS of Troina (Enna, Sicily), agreed to invite John Opitz to visit the Sicilian Pediatric Department each year. The purpose was to deliver lectures and examine children with rare, unknown syndromes. We were pleased to learn of his enthusiastic acceptance (Fig. 1).

At the initial meeting, John Opitz’s towering stature and stern demeanor initially piqued the curiosity and provoked the unease of the students. However, upon hearing his first words, his profound humanity, courtly demeanor, and engaging speech, characterized by a low, hoarse voice and carefully chosen words, became apparent. What struck the students was the simplicity and naturalness of his lectures, along with his friendly demeanor. Opitz’s clinical examinations of the children were unique, showcasing his high clinical competence, scrupulous examination skills, and graceful approach to interacting with both children and parents. He began by brightening the children’s spirits with smiles and caresses before engaging the parents in discussions about family history, concerns, and the child’s symptoms. His physical examinations commenced with observing the hands for dermatoglyphics, followed by an assessment for malformations throughout the body, and concluded with a thorough evaluation of neurological and cognitive aspects. Opitz delivered diagnoses with gentleness, aiming to alleviate parental stress.

John Opitz’s teaching significantly contributed to the establishment of the School of Clinical Genetics in Sicily, particularly in Palermo, Messina, and Catania. In 2015, he was invited to the 43rd Sicilian Congress of Pediatrics to deliver a Master’s Lecture, which, due to health reasons, he presented via video conference on the theme “Storytelling in Pediatrics and Genetics: Lessons from Aesop and Mendel,” later published in this Journal. [2].

## Conclusions

John Opitz was a man of profound humanity and diverse cultural interests, encompassing historical medicine, music, literature, human evolution, and antiquities. His fascination with Sicilian culture and history was evident during his visits, where he expressed awe at the beauty of the Temples in Agrigento and spent hours examining mummified bodies at the Museum of the “Cappuccini” Cemetery in Palermo. Despite encountering unforeseen obstacles, such as a failed visit to the State Historical Archive in Catania, Opitz’s passion for history remained undiminished.

Several episodes highlight the simplicity and friendship of this remarkable scientist. For instance, upon traveling to Sicily, John’s tall stature posed challenges, such as fitting comfortably on airplanes, which we resolved by arranging first-class tickets and accommodating him in hotels or apartments by providing two beds, with the second-placed transversally. Another instance occurred during a two-hour journey from Catania to Palermo in a small car, where he endured discomfort curled up due to his long legs, yet he didn’t complain upon arrival. On a separate occasion, he was asked to visit and offer advice to the parents of a child with autism, whose father was a wealthy and influential politician. Despite the visit lasting over three hours and concluding without expressions of gratitude or payment, John remained unperturbed, displaying his remarkable refinement and graciousness.

*To remember John Opitz: two words*,* a man and a myth.*

## Data Availability

Not applicable.
